# Intestinal pathogens detected in cockroach species within different food-related environment in Pudong, China

**DOI:** 10.1038/s41598-024-52306-x

**Published:** 2024-01-23

**Authors:** Jun Liu, Yongting Yuan, Lei Feng, Chen Lin, Chuchu Ye, Jingyi Liu, Huihui Li, Lipeng Hao, Hanzhao Liu

**Affiliations:** 1 Pudong New Area Center for Disease Control and Prevention, Shanghai, 200136 China; 2 Fudan University Pudong Institute of Preventive Medicine, Shanghai, 200136 China

**Keywords:** Entomology, Pathogens

## Abstract

Cockroaches are considered mechanical transmitters of infectious diseases, posing a threat to human health. This study assessed the potential of cockroaches in food-related environments to mechanically transmit intestinal pathogens. Cockroaches captured with traps were placed together into a low temperature refrigerator at − 80° for 2 h. Standard taxonomic keys and Fluorescent quantitative PCR techniques were applied for species identification and digestive tract etiological examination. A total of 360 cockroach traps were placed, with a positive rate of 20.8%, and 266 cockroaches were captured. In general, compared with other places and areas, the degree of infestation of cockroaches was more serious in catering places and kitchens. *Blattella germanica* were most found in catering places (40.2%), followed by *Periplaneta fuliginosa* in schools (22.2%). According to the life stage, among the 128 cockroach samples, 23 were positive for nymphs and 13 were positive for adults. There were statistically significant differences in the intestinal pathogen detection rates between nymphs and adults (*P* < 0.05). A total of eight intestinal pathogens were detected, and enterovirus infections were the main ones, with sapovirus being the most detected in *Blattella germanica* or nymph. Shiga toxin-producing *Escherichia coli* (STEC) was the most frequently isolated bacterium. *Blastocystis hominis* had the highest isolation rate. In contrast, 12 diarrhoeal disease pathogens were isolated, and the viruses and bacteria with the highest frequencies were norovirus and *E. coli*, respectively; no parasites were found. *Blattella germanica* and *Periplaneta fuliginosa* in food-related environments can act as potential vectors for the spread of intestinal pathogens and may pose a significant threat to public health.

## Introduction

Intestinal infectious diseases (IIDs) are a group of diseases transmitted through the digestive tract caused by bacteria, viruses, parasites and other pathogens, with fever and diarrhoea as the main symptoms^1^. The incidence of these diseases is high even in developed countries^2^. Estimates by Tam et al.^3^ suggest approximately 25% of the UK population suffer an episode of IID each year, with annual costs to the economy, population and national health services an estimated at £1.5 billion a year, according to Food Standards Agency^4^. The national data of notifiable infectious diseases in 2020 also showed that the incidence rate of infectious diarrhoea was 76.33 cases/100,000^5^. Under the strict public health measures for containing COVID-19 transmission, the incidence of common intestinal infectious diseases in China in 2020 was lower than that in the same period in 2019^5^, but they are still among infectious diseases with the highest health burden across in the world.

As a synanthropic insect, cockroaches are commonly found in places associated with human food, such as restaurants, kitchens and others. More and more studies reveal that their infestation trend in human dwelling environments is increasing which may greatly promote the spread of foodborne diseases^6^. The feeding mechanism and dirty breeding habits of cockroaches make them the most likely mechanical carriers for various human intestinal pathogens (fungi, bacteria, viruses and parasites)^7,8^. Various pathogenic microorganisms have been isolated from cockroaches, including bacteria^9–12^ (e.g., *Enterobacter*, *Pseudomonas* sp., *Staphylococcus* sp. and *Enterococcus* sp.), viruses^13,14^ (e.g., Rotavirus and Enterovirus), various fungi^15–17^ (e.g., *Candida* spp., *Penicillium* spp., *Aspergillus* spp. and *Acremonium* spp.) and parasites^13,18,19^ (e.g., hookworm, *Cryptosporidium* spp., *Entamoeba histolytica*, *Cyclospora* spp.). The vast majority of them are intestinal pathogens, with bacteria as the main pathogens. However, studies on cockroach and related pathogens in hospitals^9,12,20^ and residential areas^21,22^, but there are few relevant studies in catering places.

Cockroaches prefer to live in a variety of habitats, especially in warm, dark, humid and food-abundant places^18,23^. Furthermore, they are omnivores and eat any organic food but prefer food sources such as sweets, meat products, starches and grease. They also feed on plants, vegetables and fruits^24^, and catering places are ideal breeding grounds. The body parts (appendages, mouthparts, antennae) and secretions of cockroaches are the way they carry pathogens^12^. The national cockroach surveillance report in China in 2019 shows that among the various areas monitored across the country, the density of cockroaches in catering places is the second highest, only lower than that of the farmers' markets^25^. The market sewers, drains and damp rotting environments have also become habitats for cockroaches^18^. The prevalence of cockroaches near human and animal excreta, human food and the environment has drawn considerable attention to their role as vectors^26^, but there is still a lack of conclusive evidence that cockroaches are vectors of enteric infectious diseases in human.

Studies in China and abroad have shown that cockroaches caught in catering places carried different foodborne bacterial pathogens, such as *Salmonella*, *Escherichia coli*, *Staphylococcus aureus* and *Bacillus cereus*^27^, and fungi dominated by *Candida albicans*^28^. Mould, *E. coli, Pseudomonas aeruginosa* and hepatitis B virus have been detected in imported and local cockroaches in Shanghai^29^, but there is no relevant research on intestinal pathogens carried by cockroaches in catering places in the city. Cockroach may mechanically transmit pathogens through physical displacement, reflux or faecal particles deposited onto or into exposed human food, which may be ready-to-eat or improperly cooked^23^. With the rise of the catering takeout industry, cockroaches can not only spread pathogens by polluting takeaway boxes but also rely on takeaway boxes to spread cockroach egg pods or nymphs to residents or offices, leading to the spread of cockroach breeding and new public health problems.

Foodborne diseases are a widespread and escalating public health problem worldwide. They also harbour a variety of pathogenic microorganisms, approximately a quarter of which are foodborne pathogens, so cockroaches may be important reservoirs and mechanical vectors for foodborne pathogens. In general, the role of cockroaches in human infections is poorly understood. This study aimed to assess the extent of cockroach infestation in different food-related environments in Pudong New Area of Shanghai in China, and explore the prevalence of intestinal pathogens in various cockroach species.

## Results

### Distribution and abundance of cockroaches

From April 2021 to March 2022, a total of 360 traps were placed, with a positive rate of 20.8%, and 266 cockroaches were captured. Two species of cockroach, *Blattella germanica* and *Periplaneta fuliginosa* were identified. Almost all species were found in every survey place and area. Regardless of the capture places or areas, the most prevalent species was *Blattella germanica* (65.8%), followed by *Periplaneta fuliginosa* (34.2%) (Table 1, and Database: Supplementary Information 1). Among the captured places, *Blattella germanica* had the highest prevalent in catering place (40.2%), followed by *Periplaneta fuliginosa* in school (22.2%). From the perspective of captured areas, both species of cockroaches were most prevalent in kitchens (*Blattella germanica* 50.0%, *Periplaneta fuliginosa* 26.7%), compared with handling rooms and storage rooms. Additionally, according to the life stages of cockroaches, 158 nymphs and 108 adults were identified. Both nymph and adult was more prevalent in catering place and kitchen area (Table 2, and Database: Supplementary Information 1).

**Table 1:** Distribution of cockroach species by capture places and areas.

Sampling points	Places/areas	Cockroach species n (%)		Total
		*Blattella germanica*	*Periplaneta fuliginosa*	
Captures places	Catering place	107 (40.2)	8 (3.0)	115 (43.2)
	School	10 (3.8)	59 (22.2)	69 (26.0)
	Enterprises and institutions	58 (21.8)	24 (9.0)	82 (30.8)
Total		175 (65.8)	91 (34.2)	266 (100)
Captures areas	Kitchen	133 (50.0)	71 (26.7)	204 (76.7)
	Handling room	30 (11.3)	13 (4.9)	43 (16.2)
	Storage room	12 (4.5)	7 (2.6)	19 (7.1)
Total		175 (65.8)	91 (34.2)	266 (100)

**Table 2:** Distribution of cockroach nymph and adult by capture places and areas.

Sampling points	Places/areas	Life stages n (%)		Total
		Nymph	Adult	
Captures places	Catering place	59 (22.2)	56 (21.1)	115 (43.2)
	School	50 (18.8)	19 (7.1)	69 (26.0)
	Enterprises and institutions	49 (18.4)	33 (12.4)	82 (30.8)
Total		158 (59.4)	108 (40.6)	266 (100)
Captures areas	Kitchen	120 (45.1)	84 (31.6)	204 (76.7)
	Handling room	29 (10.9)	14 (5.3)	43 (16.2)
	Storage room	9 (3.3)	10 (3.8)	19 (7.1)
Total		158 (59.4)	108 (40.6)	266 (100)

### Types of intestinal pathogens detected from cockroaches digestive tract

A total of 128 cockroach samples were obtained, of which 36 were positive. Among all cockroaches carried with intestinal pathogens, 44.4% only had viruses, which was higher than those only with bacteria and parasites, and 13.9% carried all types of intestinal pathogens. In different life stages, the detection rate of intestinal pathogens in cockroach nymphs was higher than that of adults (*P* < 0.05), mainly viruses and parasites (Table 3, and Database: Supplementary Information 2).

**Table 3:** The type of intestinal pathogens detected in 128 cockroach samples.

Category	Samples of cockroaches n (%)		*P*	Types of pathogens n (%)			
	N	Positive		Only viruses	Only bacteria	Only parasites	Mixed infection
All	128	36 (28.1)		16 (44.4)	6 (16.7)	9 (25.0)	5 (13.9)
Place							
Catering place	54	19 (35.2)	> 0.05	7	0	9	3
School	39	10 (25.6)		5	5	0	0
Enterprises and institutions	35	7 (20.0)		4	1	0	2
Specie							
*Blattella germanica*	76	24 (31.6)	> 0.05	10	0	9	5
*Periplaneta fuliginosa*	52	12 (23.1)		6	6	0	0
Life stage							
Nymph	59	23 (39.0)	< 0.05	10	3	6	4
Adult	69	13 (18.8)		6	3	3	1

< 0.05, Chi-square test result was statistical different.

### Species of intestinal pathogens identified

**Figure 1:**
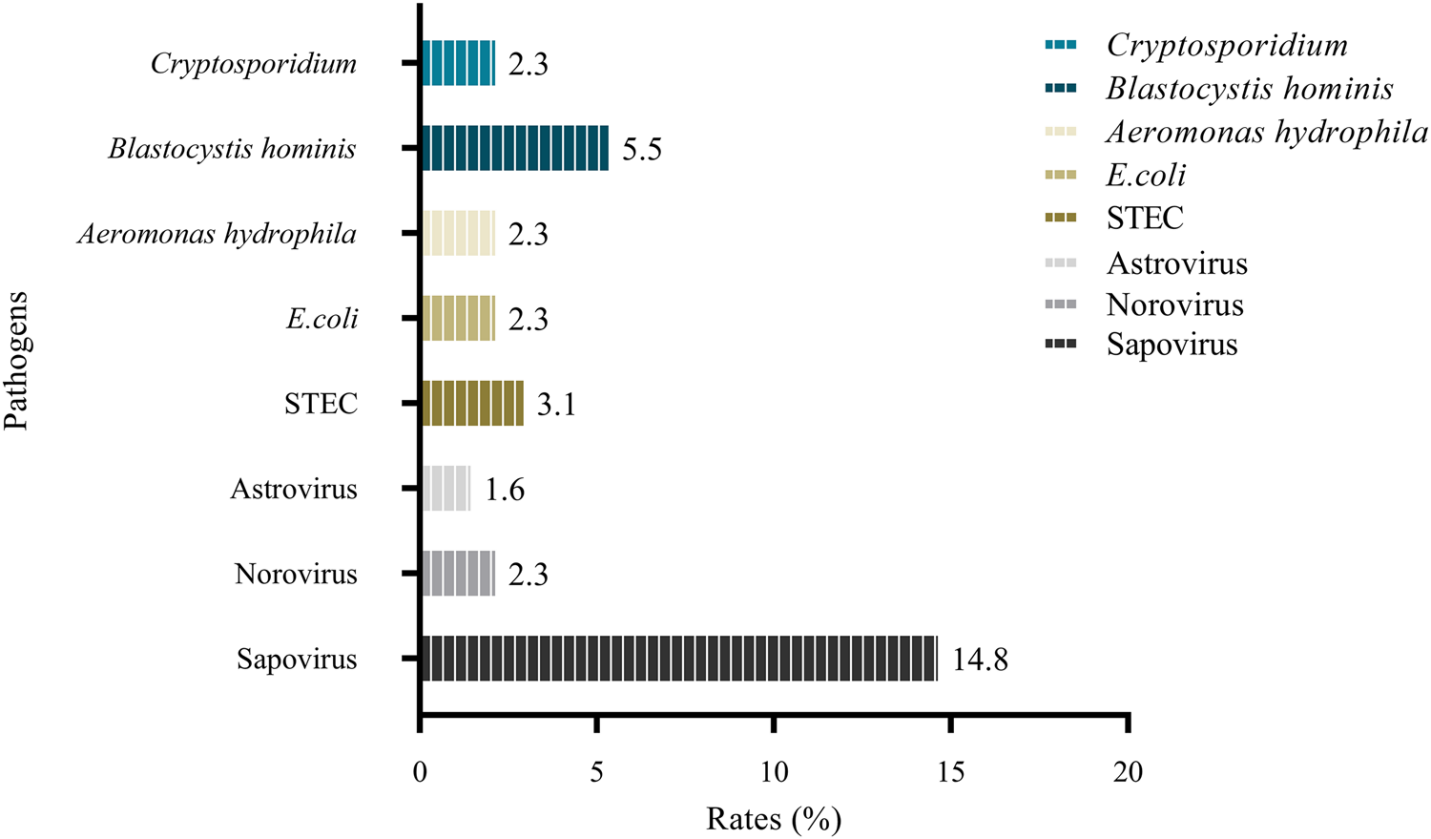
Composition and rate of pathogens detected in cockroaches.

It was found that a total of 8 species (44 stains) of intestinal pathogens were isolated (Fig. 1). The most common pathogen were viruses, of which sapovirus (19/128) had the highest detection rate, followed by norovirus (3/128) and astrovirus (2/128). Bacteria detected in cockroaches were STEC (4/128), *E. coli* (3/128) and *Aeromonas hydrophila* (3/128). *Cryptosporidium* and *Blastocystis hominis* were also found in cockroaches, with detection rates of 5.5% and 2.3%, respectively (Database: Supplementary Information 2).

### Life stages and species-related infestation of cockroaches

As presented in Table 4, compared to *Periplaneta fuliginosa* (12/52), all intestinal pathogens identified were more prevalent in *Blattella germanica* (32/76). Sapovirus (18.4%) had the highest prevalent in both species of cockroach. *E. coli* and *Aeromonas hydrophila* were not detected in *Blattella germanica*, and norovirus, STEC and parasite were not detected in *Periplaneta fuliginosa*. In terms of the life stages, nymphs (30/59) appeared to have more intestinal pathogens than adults (14/69). Sapovirus also had the highest prevalent in nymphs (20.3%) and adults (10.1%). *Aeromonas hydrophila* was not detected in nymphs, and norovirus, astrovirus, *E. coli* and *Cryptosporidium* were not found in adults (Database: Supplementary Information 2).

**Table 4:** Prevalence of intestinal pathogens grouped by species and life stages of cockroaches.

Pathogens	Cockroach species n (%)		Life stages n (%)	
	*Blattella germanica* (n = 76)	*Periplaneta fuliginosa* (n = 52)	Nymph (n = 59)	Adult (n = 69)
Virus				
Sapovirus	14 (18.4)	5 (9.6)	12 (20.3)	7 (10.1)
Norovirus	3 (3.9)	0 (0.0)	3 (5.1)	0 (0.0)
Astrovirus	1 (1.3)	1 (1.9)	2 (3.4)	0 (0.0)
Bacteria				
*E. coli*	0 (0.0)	3 (5.8)	3 (5.1)	0 (0.0)
*Aeromonas hydrophila*	0 (0.0)	3 (5.8)	0 (0.0)	3 (4.3)
STEC	4 (5.3)	0 (0.0)	3 (5.1)	1 (1.4)
Parasite				
*Cryptosporidium*	3 (3.9)	0 (0.0)	3 (5.1)	0 (0.0)
*Blastocystis hominis*	7 (9.2)	0 (0.0)	4 (6.8)	3 (4.3)
Total	32 (42.1)	12 (23.1)	30 (50.8)	14 (20.3)

*E. coli, Escherichia coli*; STEC, Shiga toxin-producing *E. coli.*

### Intestinal pathogens detection rate from cockroaches in different captured places

The study revealed that cockroaches trapped from different locations (catering places, schools, and enterprises and institutions) shared different intestinal pathogens. The detection rate of sapovirus was the highest among cockroaches caught in these places, and other viruses and bacteria were also detected. Parasites were only found in the cockroaches caught in catering places (Table 5, and Database: Supplementary Information 2).

**Table 5:** Prevalence of intestinal pathogens grouped by captured places.

Pathogens	Capture places n (%)		
	Catering places n = 54	Schools n = 39	Enterprises and institutions n = 35
Virus			
Sapovirus	9 (16.7)	4 (10.3)	6 (17.1)
Norovirus	2 (3.7)	0 (0.0)	1 (2.9)
Astrovirus	1 (1.9)	1 (2.6)	0 (0.0)
Bacteria			
*E. coli*	0 (0.0)	3 (7.7)	0 (0.0)
*Aeromonas hydrophila*	0 (0.0)	2 (5.1)	1 (2.9)
STEC	2 (3.7)	0 (0.0)	2 (5.7)
Parasite			
*Cryptosporidium*	3 (5.6)	0 (0.0)	0 (0.0)
*Blastocystis hominis*	7 (13.0)	0 (0.0)	0 (0.0)
Total	24 (44.4)	10 (25.6)	10 (28.6)

*E. coli, Escherichia coli*; STEC, Shiga toxin-producing *E. coli.*

### Species diversity of intestinal pathogens in cockroaches and humans

**Figure 2:**
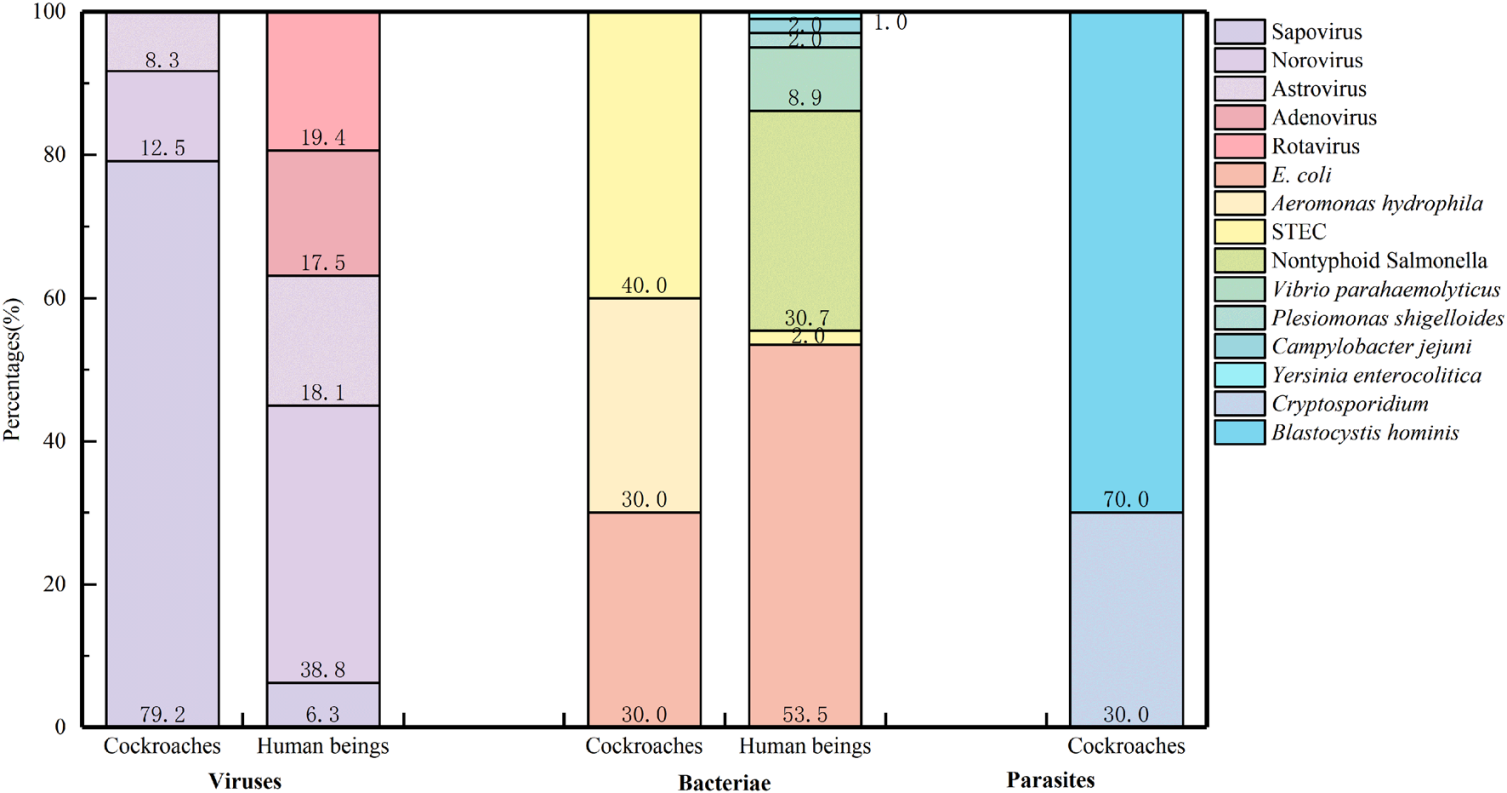
Comparison of intestinal pathogen detection between cockroaches and human beings.

As shown in Fig. 2, there were a total of eight pathogens in the intestinal pathogen spectrum of cockroaches, and 12 pathogens from the comprehensive surveillance of human diarrhoea disease were isolated. In terms of viruses, Sapovirus came in the first position in terms of isolate frequency, with 19 isolated strains (79.2%). The second and the third most common viruses were norovirus and astrovirus, with relative frequencies of 12.5% and 8.3%, respectively. In contrast, the most commonly isolated virus from human diarrhoeal disease surveillance was norovirus, with a relative frequency of 38.8%. Rotavirus and astrovirus were found to be the second and third most abundant viruses, with relative frequencies of 19.4% and 18.1%, respectively. Bacteria detected in cockroaches were E. coli (30.0%), Aeromonas hydrophila (30.0%) and STEC (40.0%). Human diarrhoeal disease surveillance has also detected E. coli (53.5%), nontyphoid *Salmonella* (30.7%), Vibrio parahaemolyticus (8.9%), *Plesiomonas shigelloides* (2.0%), Campylobacter jejuni (2.0%) and Yersinia enterocolitica (1.0%). *Cryptosporidium* and Blastocystis hominis were mainly detected in cockroaches, with relative frequencies of 30.0% and 70.0%, respectively. No parasite was found in human diarrhoeal disease surveillance (Database: Supplementary Information 2 and 3).

**Figure 3:**
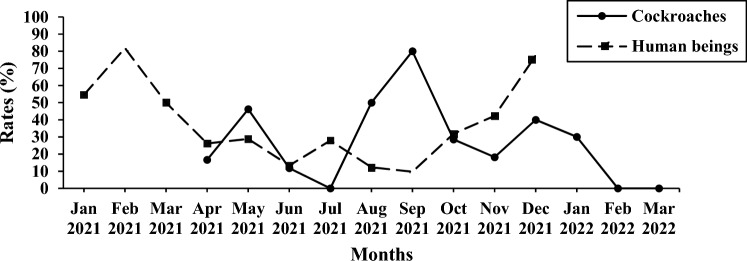
Comparison of the monthly distribution of intestinal pathogens detection rates between Cockroaches and Human beings.

From the perspective of the time change trend, the peak and change trend of intestinal pathogens were different between cockroaches and humans. The detection peak of cockroaches was in September, and it was relatively low in other months, while the detection peak of human intestinal pathogens was mainly in winter, spring and summer (Fig. 3, and Database: Supplementary Information 2 and 3).

## Discussion

Cockroaches are one of nuisance pests whose activities negatively affect humans. Of great concern to human and public health is their potential as mechanical vectors of the spread of infectious diseases, including intestinal pathogens. Previous studies from areas of Iran^30^, Algeria^31^, Thailand^18^ and elsewhere^27^ reported that cockroaches captured from residential dwellings, hospitals and fresh markets carried a range of intestinal pathogens. Due to the feeding habits of cockroaches and their preference for human food, catering places are more likely to become an environment for cockroaches to spread intestinal diseases. This study found that cockroaches captured in food handling areas carried viruses, bacteria and parasites, indicating that their potential and/or role as mechanical vectors of infectious diseases cannot be ignored.

The cockroach species captured from the study site were identified as *Blattella germanica and Periplaneta fuliginosa*, which were among the most common species in China^32^. This could be mainly due to their worldwide distribution and to their ability to breed and reproduce more easily in subtropical monsoon climates. Previous studies in Shanghai had also shown that they were the dominant populations^33,34^. *Blattella germanica* was the most frequently trapped in food-related environment as compared to *Periplaneta fuliginosa*. *Blattella germanica* has a high reproductive potential^35^, and they reproduce faster than other cockroaches growing from eggs to reproductive adults^36^, which may result in *Blattella germanica* outnumber *Periplaneta fuliginosa*. Cockroaches were ecologically classified as domestic, peridomestic, or feral. Domestic species such as *Blattella germanica*, rely heavily on resources from humans rather than surviving outdoors. Peridomestic species does not depend on humans for survival, but their proximity to human settlements made them adept at taking advantage of the amenities of civilization^35^. *Periplaneta fuliginosa* belongs to this species.

The physiological habits of cockroaches determine their habit preferences living in or around buildings. We found that *Blattella germanica* was more prevalent in catering place, whereas *Periplaneta fuliginosa* was higher in school. Studies have shown that *Periplaneta fuliginosa* prefer dark, warm and humid environments. At outdoors, they mainly focus on habitats associated with tree piles and leaf piles. Inside buildings, they also seek ecologically equivalent areas such as panels and storage areas^35^. Food service areas in schools were more likely to have conditions conducive to the breeding of cockroaches^37^. This may be due to negative phototaxis, which leads them to prefer plant environments^38,39^. The contour map generated by kriging by Brenner et al.^40^ showed the importance of tree holes. The green spaces in schools may be harborages, cockroach forage for food and water, generally returning to harbourages. *Blattella germanica* can survive well in any human habitat that provides warm moisture and food, and is mainly found in kitchens and pantries^35^. In Malaysia and China, this species has been reported as a common restaurant pest and was the dominant species trapped in restaurants in five areas of Kuala Lumpur, Malaysia, accounting for 91.1%^41^.

The degree of cockroach damage varies in different food handling areas, as shown in our study that both species of cockroaches were more prevalent in kitchens. This result is consistent with the findings of Solomon et al. in Ethiopia^27^ but it is different with the of Zha et al.^42^. That traps placed near stoves and refrigerators caught significantly more cockroaches than traps in kitchens of apartments. This might be due to the differences in the building structure. Regardless of nymphs or adults, their prevalence places and areas seem to be the same, concentrated in catering places and kitchens.

Routine investigations on IID abroad include *salmonellosis*, *shigellosis*, *campylobacteriosis*, rotavirus, norovirus and parasitic infections, but more than half of laboratory investigations of diarrhea have yet to identify a cause^43^. It is already a scientific hypothesis that pathogens can be also transmitted by domesticated insects living around humans. Studies have shown that insects such as cockroaches and houseflies living in sewage systems are mechanical carriers of viruses, bacteria and parasites^44–46^. In the present study, human enteroviruses, including sapoviruses, noroviruses and astroviruses, were detected from cockroaches in food-related environments, which were also found in human diarrhoeal disease surveillance. Research found that the common pathogens of infectious diarrhea in children include salmonella, rotavirus, shigella, vibrio, and norovirus^47^. The types of viruses detected in the above study are similar to those in this study. The two most prevalent pathogens found in diarrheal disease surveillance are norovirus and rotavirus, both of which are common causative agents, of viral diarrhoea. Norovirus is a highly contagious enterovirus that is easily spread through indirect contact with food raw materials^48^. Although rotavirus was not found in cockroaches in our study, Patience et al. detected rotavirus in cockroaches captured in the hospitals^13^, suggesting that cockroaches might spread rotavirus in various settings. In a ranking on the risk of foodborne microbial hazards, norovirus cases were included in infectious or toxic diseases caused by the consumption of food or water^49^. Cockroaches can encounter human food and food production sites, and their role in the spread of diseases is undeniable.

Existing studies have found that cockroaches can carry about 50 species of pathogenic bacteria, of which STEC, *Campylobacter* and *Salmonella* species are often thought to be linked to outbreaks of foodborne illness^50^. A meta-analysis found that cockroaches caught in the food establishment were contaminated by *E. coli*, *Klebsiella pneumoniae* and *Salmonella* spp., and that human infection with these bacteria might be transmitted through cockroaches^51^. In this study, three species of bacteria, *E. coli*, *Aeromonas hydrophila* and STEC, were detected in cockroaches. *E. coli* has also been found in food processing sites in Ethiopia. *E. coli* O157, a microorganism that lives in the intestines of live animals and is excreted through their faeces, potentially contaminating food, water and the environment, was isolated from cockroaches^52,53^. The STEC strain can survive harsh food processing conditions and has been a hot spot for persistent cross-contamination incidents in food processing environments^50^. Aeromonas species are found in aquatic environments, food, vertebrates and invertebrates, ticks and insects, of which *Aeromonas hydrophila* is believed to be pathogenic and clinically significant to humans^54^.

Although there is a lack of extensive investigation, the variety of environmental sources of Aeromonas can easily lead to constant contact and interaction with humans. Salmonella and shigella infections are usually caused by eating undercooked meat and eggs and other raw products contaminated with agents, cockroaches and flies^55,56^. *Salmonella* is the most common cause of food-borne outbreaks of the enterobacterium genus in Spain, with 4420 cases of non-typhoid/paratyphoid salmonella and 36 cases of typhoid/paratyphoid Salmonella. Poultry meat contaminated with *Campylobacter jejuni* is the main route of human infection and has a high prevalence in poultry^57^. Four subspecies of *Campylobacter jejuni* have also been isolated from cockroaches caught near kitchens and poultry sheds, suggesting that cockroaches may be a potential vector for campylobacter transmission to human food^58^. Therefore, these findings suggest that cockroaches and their control may be even more important for preventing infections than currently recognized. More researches are needed to better understand the circularity and biological mechanisms of vector transmission via cockroaches in important places, such as kitchens, to minimize the exposure of cockroaches to unsanitary places/substrates from which pathogens are acquired.

Cockroaches often feed on human feces, and hence can spread intestinal parasite cysts mechanically in the environment^59^. The intestinal parasites detected in this study include *Cryptosporidium* (2.3%) and *Blastocystis hominis* (5.5%). But compared to Dokmaikaw et al.’ results^18^, the prevalence of *Cryptosporidium* was 15.4%, and *Blastocystis hominis* was 6.6%). This difference in detection rates may be due to difference in survey places and parasite identification methods. In addition, the infectivity of Sarcocystis oocysts to American cockroaches lasted for at least 20 days and to German cockroaches for 5 days after exposure to contaminated faeces^7^.

There were some limitations in this study, the detection of some intestinal pathogens by microfluidic chip was not as accurate as bacterial culture and virus isolation. Secondly, the sample size of this study is small and the time is short, and the population detection in the same place/time cannot be carried out simultaneously. In addition, only cockroaches in food related environment were collected in the study, which cannot represent the overall exposure to cockroaches in the human world. In the future, we will carry out the detection and analysis of food contamination and environmental pollution around *Blattella germanica* and *Periplaneta fuliginosa*. In other words, in a specific environment, cockroaches carrying pathogens will be tested to sample the food they have bitten and the environment they have lived in, so as to detect the direct pollution to food and environment.

## Conclusions

Cockroaches can harbour and spread many foodborne microbial pathogens, including bacteria, viruses and parasites, which means that cockroaches may play a wide range of roles in the spread of foodborne infections. Given the link between cockroaches and foodborne pathogens, it is important to consider them in foodborne outbreak investigations, which thus far have not been the case. Considering the foodborne risks associated with cockroaches, their presence in the food-related environment should not be tolerated. Therefore, it is necessary to improve the existing kitchen and environmental hygiene standards to minimize the exposure of cockroaches to unsanitary places/substrates from which pathogens are acquired.

## Materials and methods

### Study area and trapping of cockroaches

**Figure 4:**
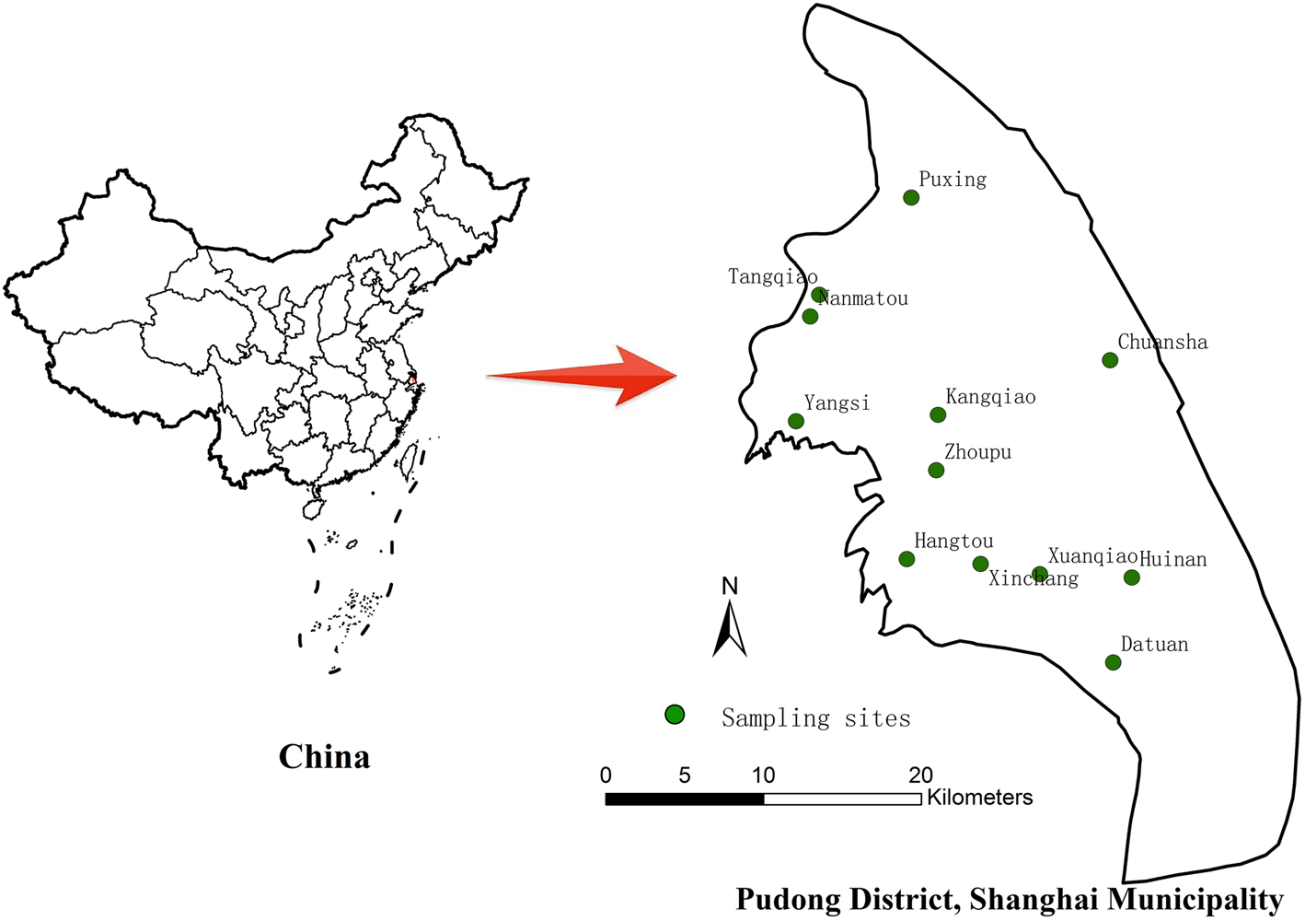
Map showing of study area in Pudong New Area, Shanghai, in Eastern China.

A longitudinal study was conducted in 12 streets and towns from April 2021 to March 2022 under the supervision of the Pudong New Area Center for Disease Control and Prevention in Shanghai (Fig. 4). Three types of places were selected to survey for each street or town, including catering places (one large restaurant, area ≥ 300 m^2^; one small restaurant, area ≤ 150 m^2^), two schools, and one enterprise and institution. One cockroach trap (made of PP, Wenzhou Oukele Biotechnology Company) containing 5 g fresh bread crumbs was placed every 15 m^2^ in the kitchens, handling rooms and storage rooms of various survey places. The traps were placed before evening and retrieved the next morning. During the investigation, 10 monitoring point hospitals in Pudong New Area, including Renji Hospital, Sixth People's Hospital, Seventh People's Hospital, Gongli Hospital, Pudong Hospital, Zhoupu Hospital, Pudong New Area People’s Hospital, Nicheng Community Hospital, Shanghai East Hospital and Yangsi Hospital, will collect diarrhoeal disease specimens and send them to Pudong New Area Center for Disease Control and Prevention for microbial testing.

### Identification of cockroaches and etiological examination

Using sterile gloves, the captured cockroaches were placed together with the trap in a − 80 ℃ cryogenic freezer for 2 h and then removed and placed on an ice steak. All collected cockroaches were examined under an anatomical microscope and identified by using standard taxonomic keys^60,61^. After identification, *Blattella germanica* captured in the same place was taken as one sample for every 5 nymphs or 2 adults, and *Periplaneta fuliginosa* was taken as one sample for every 2 nymphs or 1 adult. The samples were placed into centrifuge tubes containing 20 ml 0.9% sterile physiological saline and shaken at low speed for 5 min to remove pathogens attached to the body surface. The washed samples were shredded in a grinding tube to extract pathogens from the digestive tract, and 1 ml of Hank's solution and 2 grinding beads were added for grinding using a high-throughput tissue grinder (1 min, 60 Hz). After milling, the homogenate was transferred to a cryopreservation tube and centrifuged for 4 min (2000 r/min) at 4 ℃. After centrifugation, 200 µl of supernatant was used for nucleic acid extraction, and the remaining supernatant was stored for later use at – 80 °C.

Nucleic acids were extracted by an automatic nucleic acid extractor, mixed with premixed solution, and then added to gastrointestinal infection microfluidic chip V3. Real-time fluorescent quantitative reverse transcriptase polymerase chain reaction (RT‒PCR) was carried out using QuantStudio7.

Qualitative detection of 30 intestinal pathogens, including 18 kinds of intestinal bacterias, such as Enteroaggregative *E. coli* (EAEC), Enterohemorrhagic *E. coli*(EHEC), Enteroinvasive *E. coli* (EIEC), Enteropathogenic *E. coli*(EPEC), Enterotoxigenic *E. coli* (ETEC), Shiga toxin-producing *E. coli* (STEC), *Vibrio parahaemolyticus*, *Vibrio Vulnificus*, *Vibrio cholerae*, *Aeromonas hydrophila*, *Plesiomonas shigelloides*, *Yersinia enterocolitica*, *Campylobacter jejuni*, *Campylobacter coli*(*C. coli*), *Campylobacter upsaliensis*, *Clostridium difficile*, *Salmonella and Shigella*, 6 kinds of intestinal viruses, such as Norovirus, Astrovirus, Sapovirus, Adenovirus, Rotavirus and Parechovirus, 6 kinds of intestinal parasites, such as *Blastocystis hominis*, *Cryptosporidium*, *Dientamoeba Fragilis*, *Entamoeba histolytica*, *Cyclospora cayetanensis* and *Giardia lamblia*.

### Statistical analysis

Statistical Package for Social Sciences (SPSS) for Windows version 18.0 was used for data analysis. Descriptive statistics were used to determine prevalence, frequency, and percentage. The relationship between variables was analysed using the chi-square test. *P* < 0.05 was considered statistically significant (Supplementary Information files 1, 2 and 3).

### Supplementary Information

Supplementary Information 1.

Supplementary Information 2.

Supplementary Information 3.

## Data Availability

All data generated or analysed during this study are included in this published article (and its Supplementary Information files).
