# Supplementary material for: Intestinal pathogens detected in cockroach species within different food-related environment in Pudong, China
Source: Sci Rep. 2024 Jan 23;14:1947. doi: 10.1038/s41598-024-52306-x (PMC10803747; doi:10.1038/s41598-024-52306-x)
Supplement: Supplementary file 1 — Supplementary Information 1. [file 41598_2024_52306_MOESM1_ESM.docx]

**Database 1**

**Pathogen detection status**

| **ID** | **Month** | **Community** | **Place** | **Species** | **Development stages** | **Detection result  of Sample** | **Sapovirus (I,II,IV)** | **Sapovirus (I,II,IV&V)** | **Norovirus** | **Astrovirus** | **E.coli** | **Aeromonas Hydrophila** | **STEC** | **Cryptosporidium** | **Blastocystis Hominis** |
| --- | --- | --- | --- | --- | --- | --- | --- | --- | --- | --- | --- | --- | --- | --- | --- |
| 1 | 4 | kangqiao | Enterprises and institutions | B. germanica | Adult | Negative | Negative | Negative | Negative | Negative | Negative | Negative | Negative | Negative | Negative |
| 2 | 4 | kangqiao | Enterprises and institutions | B. germanica | Adult | Negative | Negative | Negative | Negative | Negative | Negative | Negative | Negative | Negative | Negative |
| 3 | 4 | kangqiao | Enterprises and institutions | B. germanica | Nymph | Negative | Negative | Negative | Negative | Negative | Negative | Negative | Negative | Negative | Negative |
| 4 | 4 | kangqiao | Enterprises and institutions | B. germanica | Nymph | Positive | Negative | Positive | Negative | Negative | Negative | Negative | Negative | Negative | Negative |
| 5 | 4 | kangqiao | Enterprises and institutions | B. germanica | Adult | Negative | Negative | Negative | Negative | Negative | Negative | Negative | Negative | Negative | Negative |
| 6 | 4 | kangqiao | Enterprises and institutions | B. germanica | Nymph | Negative | Negative | Negative | Negative | Negative | Negative | Negative | Negative | Negative | Negative |
| 7 | 5 | chuansha | School | P. fuliginosa | Adult | Negative | Negative | Negative | Negative | Negative | Negative | Negative | Negative | Negative | Negative |
| 8 | 5 | chuansha | School | P. fuliginosa | Nymph | Negative | Negative | Negative | Negative | Negative | Negative | Negative | Negative | Negative | Negative |
| 9 | 5 | chuansha | School | P. fuliginosa | Nymph | Negative | Negative | Negative | Negative | Negative | Negative | Negative | Negative | Negative | Negative |
| 10 | 5 | chuansha | School | P. fuliginosa | Nymph | Positive | Negative | Negative | Negative | Negative | Positive | Negative | Negative | Negative | Negative |
| 11 | 5 | chuansha | School | P. fuliginosa | Nymph | Positive | Negative | Negative | Negative | Positive | Negative | Negative | Negative | Negative | Negative |
| 12 | 5 | chuansha | School | P. fuliginosa | Nymph | Positive | Negative | Positive | Negative | Negative | Negative | Negative | Negative | Negative | Negative |
| 13 | 5 | chuansha | School | P. fuliginosa | Nymph | Positive | Positive | Negative | Negative | Negative | Negative | Negative | Negative | Negative | Negative |
| 14 | 5 | chuansha | School | P. fuliginosa | Nymph | Positive | Negative | Negative | Negative | Negative | Positive | Negative | Negative | Negative | Negative |
| 15 | 5 | chuansha | School | P. fuliginosa | Nymph | Positive | Negative | Negative | Negative | Negative | Positive | Negative | Negative | Negative | Negative |
| 16 | 5 | chuansha | Enterprises and institutions | P. fuliginosa | Adult | Negative | Negative | Negative | Negative | Negative | Negative | Negative | Negative | Negative | Negative |
| 17 | 5 | chuansha | Enterprises and institutions | P. fuliginosa | Adult | Negative | Negative | Negative | Negative | Negative | Negative | Negative | Negative | Negative | Negative |
| 18 | 5 | chuansha | Enterprises and institutions | B. germanica | Adult | Negative | Negative | Negative | Negative | Negative | Negative | Negative | Negative | Negative | Negative |
| 19 | 5 | chuansha | Enterprises and institutions | B. germanica | Nymph | Negative | Negative | Negative | Negative | Negative | Negative | Negative | Negative | Negative | Negative |
| 20 | 6 | xuanqiao | School | P. fuliginosa | Nymph | Negative | Negative | Negative | Negative | Negative | Negative | Negative | Negative | Negative | Negative |
| 21 | 6 | xuanqiao | School | P. fuliginosa | Adult | Negative | Negative | Negative | Negative | Negative | Negative | Negative | Negative | Negative | Negative |
| 22 | 6 | xuanqiao | School | P. fuliginosa | Adult | Negative | Negative | Negative | Negative | Negative | Negative | Negative | Negative | Negative | Negative |
| 23 | 6 | xuanqiao | School | P. fuliginosa | Adult | Negative | Negative | Negative | Negative | Negative | Negative | Negative | Negative | Negative | Negative |
| 24 | 6 | xuanqiao | School | P. fuliginosa | Adult | Negative | Negative | Negative | Negative | Negative | Negative | Negative | Negative | Negative | Negative |
| 25 | 6 | xuanqiao | School | P. fuliginosa | Adult | Negative | Negative | Negative | Negative | Negative | Negative | Negative | Negative | Negative | Negative |
| 26 | 6 | xuanqiao | School | P. fuliginosa | Adult | Negative | Negative | Negative | Negative | Negative | Negative | Negative | Negative | Negative | Negative |
| 27 | 6 | xuanqiao | School | P. fuliginosa | Adult | Negative | Negative | Negative | Negative | Negative | Negative | Negative | Negative | Negative | Negative |
| 28 | 6 | xuanqiao | School | P. fuliginosa | Adult | Negative | Negative | Negative | Negative | Negative | Negative | Negative | Negative | Negative | Negative |
| 29 | 6 | xuanqiao | School | P. fuliginosa | Adult | Negative | Negative | Negative | Negative | Negative | Negative | Negative | Negative | Negative | Negative |
| 30 | 6 | xuanqiao | School | P. fuliginosa | Adult | Negative | Negative | Negative | Negative | Negative | Negative | Negative | Negative | Negative | Negative |
| 31 | 6 | xuanqiao | Enterprises and institutions | P. fuliginosa | Adult | Negative | Negative | Negative | Negative | Negative | Negative | Negative | Negative | Negative | Negative |
| 32 | 6 | xuanqiao | Enterprises and institutions | P. fuliginosa | Adult | Negative | Negative | Negative | Negative | Negative | Negative | Negative | Negative | Negative | Negative |
| 33 | 6 | xuanqiao | Enterprises and institutions | P. fuliginosa | Adult | Negative | Negative | Negative | Negative | Negative | Negative | Negative | Negative | Negative | Negative |
| 34 | 6 | xuanqiao | Enterprises and institutions | P. fuliginosa | Nymph | Positive | Positive | Negative | Negative | Negative | Negative | Negative | Negative | Negative | Negative |
| 35 | 6 | xuanqiao | Enterprises and institutions | P. fuliginosa | Nymph | Positive | Negative | Positive | Negative | Negative | Negative | Negative | Negative | Negative | Negative |
| 36 | 6 | xuanqiao | Enterprises and institutions | P. fuliginosa | Nymph | Negative | Negative | Negative | Negative | Negative | Negative | Negative | Negative | Negative | Negative |
| 37 | 7 | hangtou | Catering place | P. fuliginosa | Adult | Negative | Negative | Negative | Negative | Negative | Negative | Negative | Negative | Negative | Negative |
| 38 | 7 | hangtou | Catering place | P. fuliginosa | Nymph | Negative | Negative | Negative | Negative | Negative | Negative | Negative | Negative | Negative | Negative |
| 39 | 7 | hangtou | Catering place | B. germanica | Adult | Negative | Negative | Negative | Negative | Negative | Negative | Negative | Negative | Negative | Negative |
| 40 | 7 | hangtou | Catering place | P. fuliginosa | Nymph | Negative | Negative | Negative | Negative | Negative | Negative | Negative | Negative | Negative | Negative |
| 41 | 7 | hangtou | Catering place | P. fuliginosa | Nymph | Negative | Negative | Negative | Negative | Negative | Negative | Negative | Negative | Negative | Negative |
| 42 | 7 | hangtou | School | P. fuliginosa | Adult | Negative | Negative | Negative | Negative | Negative | Negative | Negative | Negative | Negative | Negative |
| 43 | 7 | hangtou | School | P. fuliginosa | Nymph | Negative | Negative | Negative | Negative | Negative | Negative | Negative | Negative | Negative | Negative |
| 44 | 7 | hangtou | School | P. fuliginosa | Adult | Negative | Negative | Negative | Negative | Negative | Negative | Negative | Negative | Negative | Negative |
| 45 | 7 | hangtou | School | B. germanica | Nymph | Negative | Negative | Negative | Negative | Negative | Negative | Negative | Negative | Negative | Negative |
| 46 | 7 | hangtou | Enterprises and institutions | P. fuliginosa | Adult | Negative | Negative | Negative | Negative | Negative | Negative | Negative | Negative | Negative | Negative |
| 47 | 7 | hangtou | Enterprises and institutions | P. fuliginosa | Nymph | Negative | Negative | Negative | Negative | Negative | Negative | Negative | Negative | Negative | Negative |
| 48 | 8 | tangqiao | Catering place | B. germanica | Adult | Negative | Negative | Negative | Negative | Negative | Negative | Negative | Negative | Negative | Negative |
| 49 | 8 | tangqiao | Catering place | B. germanica | Adult | Positive | Positive | Positive | Negative | Negative | Negative | Negative | Negative | Negative | Negative |
| 50 | 8 | tangqiao | Catering place | B. germanica | Nymph | Positive | Negative | Negative | Negative | Negative | Negative | Negative | Negative | Positive | Negative |
| 51 | 8 | tangqiao | Catering place | B. germanica | Nymph | Positive | Positive | Positive | Positive | Negative | Negative | Negative | Negative | Negative | Negative |
| 52 | 8 | tangqiao | Catering place | B. germanica | Adult | Negative | Negative | Negative | Negative | Negative | Negative | Negative | Negative | Negative | Negative |
| 53 | 8 | tangqiao | Catering place | B. germanica | Adult | Positive | Positive | Positive | Negative | Negative | Negative | Negative | Negative | Negative | Negative |
| 54 | 8 | tangqiao | School | P. fuliginosa | Nymph | Negative | Negative | Negative | Negative | Negative | Negative | Negative | Negative | Negative | Negative |
| 55 | 8 | tangqiao | School | P. fuliginosa | Nymph | Positive | Positive | Negative | Negative | Negative | Negative | Negative | Negative | Negative | Negative |
| 56 | 8 | tangqiao | School | P. fuliginosa | Nymph | Negative | Negative | Negative | Negative | Negative | Negative | Negative | Negative | Negative | Negative |
| 57 | 8 | tangqiao | School | P. fuliginosa | Adult | Negative | Negative | Negative | Negative | Negative | Negative | Negative | Negative | Negative | Negative |
| 58 | 8 | tangqiao | School | P. fuliginosa | Adult | Positive | Negative | Negative | Negative | Negative | Negative | Positive | Negative | Negative | Negative |
| 59 | 8 | tangqiao | School | P. fuliginosa | Adult | Negative | Negative | Negative | Negative | Negative | Negative | Negative | Negative | Negative | Negative |
| 60 | 8 | tangqiao | School | P. fuliginosa | Adult | Positive | Negative | Negative | Negative | Negative | Negative | Positive | Negative | Negative | Negative |
| 61 | 8 | tangqiao | School | P. fuliginosa | Nymph | Negative | Negative | Negative | Negative | Negative | Negative | Negative | Negative | Negative | Negative |
| 62 | 8 | tangqiao | School | P. fuliginosa | Nymph | Negative | Negative | Negative | Negative | Negative | Negative | Negative | Negative | Negative | Negative |
| 63 | 8 | tangqiao | Enterprises and institutions | P. fuliginosa | Adult | Positive | Negative | Negative | Negative | Negative | Negative | Positive | Negative | Negative | Negative |
| 64 | 9 | yangsi | Catering place | B. germanica | Adult | Positive | Positive | Positive | Negative | Negative | Negative | Negative | Negative | Negative | Negative |
| 65 | 9 | yangsi | Catering place | B. germanica | Adult | Positive | Positive | Negative | Negative | Negative | Negative | Negative | Positive | Negative | Negative |
| 66 | 9 | yangsi | Catering place | B. germanica | Adult | Positive | Positive | Positive | Negative | Negative | Negative | Negative | Negative | Negative | Negative |
| 67 | 9 | yangsi | Catering place | B. germanica | Adult | Positive | Positive | Negative | Negative | Negative | Negative | Negative | Negative | Negative | Negative |
| 68 | 9 | yangsi | Catering place | B. germanica | Nymph | Positive | Positive | Positive | Positive | Negative | Negative | Negative | Positive | Negative | Negative |
| 69 | 9 | yangsi | Enterprises and institutions | B. germanica | Adult | Positive | Positive | Positive | Negative | Negative | Negative | Negative | Negative | Negative | Negative |
| 70 | 9 | yangsi | Enterprises and institutions | B. germanica | Adult | Negative | Negative | Negative | Negative | Negative | Negative | Negative | Negative | Negative | Negative |
| 71 | 9 | yangsi | Enterprises and institutions | B. germanica | Adult | Negative | Negative | Negative | Negative | Negative | Negative | Negative | Negative | Negative | Negative |
| 72 | 9 | yangsi | Enterprises and institutions | B. germanica | Nymph | Positive | Positive | Positive | Negative | Negative | Negative | Negative | Positive | Negative | Negative |
| 73 | 9 | yangsi | Enterprises and institutions | B. germanica | Nymph | Positive | Positive | Positive | Positive | Negative | Negative | Negative | Positive | Negative | Negative |
| 74 | 10 | nanmatou | Catering place | B. germanica | Nymph | Negative | Negative | Negative | Negative | Negative | Negative | Negative | Negative | Negative | Negative |
| 75 | 10 | nanmatou | Catering place | B. germanica | Nymph | Negative | Negative | Negative | Negative | Negative | Negative | Negative | Negative | Negative | Negative |
| 76 | 10 | nanmatou | Catering place | B. germanica | Nymph | Positive | Positive | Negative | Negative | Negative | Negative | Negative | Negative | Negative | Negative |
| 77 | 10 | nanmatou | Catering place | B. germanica | Nymph | Positive | Negative | Negative | Negative | Negative | Negative | Negative | Negative | Positive | Negative |
| 78 | 10 | nanmatou | Catering place | B. germanica | Nymph | Negative | Negative | Negative | Negative | Negative | Negative | Negative | Negative | Negative | Negative |
| 79 | 10 | nanmatou | School | B. germanica | Nymph | Negative | Negative | Negative | Negative | Negative | Negative | Negative | Negative | Negative | Negative |
| 80 | 10 | nanmatou | School | B. germanica | Nymph | Negative | Negative | Negative | Negative | Negative | Negative | Negative | Negative | Negative | Negative |
| 81 | 11 | puxing | Catering place | B. germanica | Adult | Negative | Negative | Negative | Negative | Negative | Negative | Negative | Negative | Negative | Negative |
| 82 | 11 | puxing | Catering place | B. germanica | Adult | Positive | Negative | Negative | Negative | Negative | Negative | Negative | Negative | Negative | Positive |
| 83 | 11 | puxing | Catering place | B. germanica | Adult | Negative | Negative | Negative | Negative | Negative | Negative | Negative | Negative | Negative | Negative |
| 84 | 11 | puxing | Catering place | B. germanica | Nymph | Positive | Negative | Negative | Negative | Negative | Negative | Negative | Negative | Negative | Positive |
| 85 | 11 | puxing | Enterprises and institutions | B. germanica | Adult | Negative | Negative | Negative | Negative | Negative | Negative | Negative | Negative | Negative | Negative |
| 86 | 11 | puxing | Enterprises and institutions | B. germanica | Nymph | Negative | Negative | Negative | Negative | Negative | Negative | Negative | Negative | Negative | Negative |
| 87 | 11 | puxing | Enterprises and institutions | B. germanica | Nymph | Negative | Negative | Negative | Negative | Negative | Negative | Negative | Negative | Negative | Negative |
| 88 | 11 | puxing | Enterprises and institutions | P. fuliginosa | Nymph | Negative | Negative | Negative | Negative | Negative | Negative | Negative | Negative | Negative | Negative |
| 89 | 11 | puxing | Enterprises and institutions | B. germanica | Adult | Negative | Negative | Negative | Negative | Negative | Negative | Negative | Negative | Negative | Negative |
| 90 | 11 | puxing | Enterprises and institutions | B. germanica | Nymph | Negative | Negative | Negative | Negative | Negative | Negative | Negative | Negative | Negative | Negative |
| 91 | 11 | puxing | Enterprises and institutions | B. germanica | Nymph | Negative | Negative | Negative | Negative | Negative | Negative | Negative | Negative | Negative | Negative |
| 92 | 12 | huinan | Catering place | B. germanica | Adult | Negative | Negative | Negative | Negative | Negative | Negative | Negative | Negative | Negative | Negative |
| 93 | 12 | huinan | Catering place | B. germanica | Nymph | Positive | Negative | Negative | Negative | Negative | Negative | Negative | Negative | Negative | Positive |
| 94 | 12 | huinan | Catering place | B. germanica | Adult | Negative | Negative | Negative | Negative | Negative | Negative | Negative | Negative | Negative | Negative |
| 95 | 12 | huinan | Catering place | B. germanica | Adult | Negative | Negative | Negative | Negative | Negative | Negative | Negative | Negative | Negative | Negative |
| 96 | 12 | huinan | Catering place | B. germanica | Nymph | Positive | Negative | Negative | Negative | Negative | Negative | Negative | Negative | Negative | Positive |
| 97 | 12 | huinan | Catering place | B. germanica | Nymph | Positive | Negative | Negative | Negative | Positive | Negative | Negative | Negative | Negative | Positive |
| 98 | 12 | huinan | Catering place | B. germanica | Adult | Negative | Negative | Negative | Negative | Negative | Negative | Negative | Negative | Negative | Negative |
| 99 | 12 | huinan | Catering place | B. germanica | Nymph | Negative | Negative | Negative | Negative | Negative | Negative | Negative | Negative | Negative | Negative |
| 100 | 12 | huinan | School | B. germanica | Adult | Negative | Negative | Negative | Negative | Negative | Negative | Negative | Negative | Negative | Negative |
| 101 | 12 | huinan | School | B. germanica | Nymph | Positive | Positive | Negative | Negative | Negative | Negative | Negative | Negative | Negative | Negative |
| 102 | 1 | datuan | Catering place | B. germanica | Adult | Negative | Negative | Negative | Negative | Negative | Negative | Negative | Negative | Negative | Negative |
| 103 | 1 | datuan | Catering place | B. germanica | Adult | Negative | Negative | Negative | Negative | Negative | Negative | Negative | Negative | Negative | Negative |
| 104 | 1 | datuan | Catering place | B. germanica | Nymph | Negative | Negative | Negative | Negative | Negative | Negative | Negative | Negative | Negative | Negative |
| 105 | 1 | datuan | Catering place | B. germanica | Adult | Positive | Negative | Negative | Negative | Negative | Negative | Negative | Negative | Negative | Positive |
| 106 | 1 | datuan | Catering place | B. germanica | Adult | Negative | Negative | Negative | Negative | Negative | Negative | Negative | Negative | Negative | Negative |
| 107 | 1 | datuan | Catering place | B. germanica | Adult | Positive | Negative | Negative | Negative | Negative | Negative | Negative | Negative | Negative | Positive |
| 108 | 1 | datuan | Catering place | B. germanica | Adult | Negative | Negative | Negative | Negative | Negative | Negative | Negative | Negative | Negative | Negative |
| 109 | 1 | datuan | Catering place | B. germanica | Adult | Negative | Negative | Negative | Negative | Negative | Negative | Negative | Negative | Negative | Negative |
| 110 | 1 | datuan | Catering place | B. germanica | Nymph | Negative | Negative | Negative | Negative | Negative | Negative | Negative | Negative | Negative | Negative |
| 111 | 1 | datuan | Catering place | B. germanica | Nymph | Positive | Negative | Negative | Negative | Negative | Negative | Negative | Negative | Positive | Negative |
| 112 | 2 | xinchang | Catering place | B. germanica | Adult | Negative | Negative | Negative | Negative | Negative | Negative | Negative | Negative | Negative | Negative |
| 113 | 2 | xinchang | Catering place | B. germanica | Adult | Negative | Negative | Negative | Negative | Negative | Negative | Negative | Negative | Negative | Negative |
| 114 | 2 | xinchang | Catering place | B. germanica | Adult | Negative | Negative | Negative | Negative | Negative | Negative | Negative | Negative | Negative | Negative |
| 115 | 2 | xinchang | Catering place | B. germanica | Nymph | Negative | Negative | Negative | Negative | Negative | Negative | Negative | Negative | Negative | Negative |
| 116 | 2 | xinchang | Catering place | B. germanica | Adult | Negative | Negative | Negative | Negative | Negative | Negative | Negative | Negative | Negative | Negative |
| 117 | 2 | xinchang | Catering place | B. germanica | Adult | Negative | Negative | Negative | Negative | Negative | Negative | Negative | Negative | Negative | Negative |
| 118 | 2 | xinchang | Catering place | B. germanica | Adult | Negative | Negative | Negative | Negative | Negative | Negative | Negative | Negative | Negative | Negative |
| 119 | 2 | xinchang | Enterprises and institutions | B. germanica | Adult | Negative | Negative | Negative | Negative | Negative | Negative | Negative | Negative | Negative | Negative |
| 120 | 2 | xinchang | Enterprises and institutions | B. germanica | Nymph | Negative | Negative | Negative | Negative | Negative | Negative | Negative | Negative | Negative | Negative |
| 121 | 3 | zhoupu | Catering place | B. germanica | Nymph | Negative | Negative | Negative | Negative | Negative | Negative | Negative | Negative | Negative | Negative |
| 122 | 3 | zhoupu | Catering place | B. germanica | Nymph | Negative | Negative | Negative | Negative | Negative | Negative | Negative | Negative | Negative | Negative |
| 123 | 3 | zhoupu | Catering place | B. germanica | Adult | Negative | Negative | Negative | Negative | Negative | Negative | Negative | Negative | Negative | Negative |
| 124 | 3 | zhoupu | Catering place | B. germanica | Adult | Negative | Negative | Negative | Negative | Negative | Negative | Negative | Negative | Negative | Negative |
| 125 | 3 | zhoupu | School | P. fuliginosa | Nymph | Negative | Negative | Negative | Negative | Negative | Negative | Negative | Negative | Negative | Negative |
| 126 | 3 | zhoupu | School | P. fuliginosa | Adult | Negative | Negative | Negative | Negative | Negative | Negative | Negative | Negative | Negative | Negative |
| 127 | 3 | zhoupu | Enterprises and institutions | P. fuliginosa | Nymph | Negative | Negative | Negative | Negative | Negative | Negative | Negative | Negative | Negative | Negative |
| 128 | 3 | zhoupu | Enterprises and institutions | P. fuliginosa | Adult | Negative | Negative | Negative | Negative | Negative | Negative | Negative | Negative | Negative | Negative |
