# Supplementary material for: Intestinal pathogens detected in cockroach species within different food-related environment in Pudong, China
Source: Sci Rep. 2024 Jan 23;14:1947. doi: 10.1038/s41598-024-52306-x (PMC10803747; doi:10.1038/s41598-024-52306-x)
Supplement: Supplementary file 2 — Supplementary Information 2. [file 41598_2024_52306_MOESM2_ESM.doc]

**Database 1**

**Magnitude of cockroach infestation**

| **ID** | **Month** | **Community** | **Place** | **Area** | **Traps (Negative/Positive)** | **No.of traps** |
| --- | --- | --- | --- | --- | --- | --- |
| 1 | 4 | kangqiao | Catering place | Kitchen | Negative | 3 |
| 2 | 4 | kangqiao | Catering place | Kitchen | Positive | 0 |
| 3 | 4 | kangqiao | Catering place | Handling room | Negative | 3 |
| 4 | 4 | kangqiao | Catering place | Handling room | Positive | 0 |
| 5 | 4 | kangqiao | Catering place | Storage room | Negative | 1 |
| 6 | 4 | kangqiao | Catering place | Storage room | Positive | 0 |
| 7 | 4 | kangqiao | School | Kitchen | Negative | 7 |
| 8 | 4 | kangqiao | School | Kitchen | Positive | 0 |
| 9 | 4 | kangqiao | School | Handling room | Negative | 6 |
| 10 | 4 | kangqiao | School | Handling room | Positive | 0 |
| 11 | 4 | kangqiao | School | Storage room | Negative | 2 |
| 12 | 4 | kangqiao | School | Storage room | Positive | 0 |
| 13 | 4 | kangqiao | Enterprises and institutions | Kitchen | Negative | 3 |
| 14 | 4 | kangqiao | Enterprises and institutions | Kitchen | Positive | 2 |
| 15 | 4 | kangqiao | Enterprises and institutions | Handling room | Negative | 2 |
| 16 | 4 | kangqiao | Enterprises and institutions | Handling room | Positive | 0 |
| 17 | 4 | kangqiao | Enterprises and institutions | Storage room | Negative | 0 |
| 18 | 4 | kangqiao | Enterprises and institutions | Storage room | Positive | 1 |
| 19 | 5 | chuansha | Catering place | Kitchen | Negative | 5 |
| 20 | 5 | chuansha | Catering place | Kitchen | Positive | 0 |
| 21 | 5 | chuansha | Catering place | Handling room | Negative | 3 |
| 22 | 5 | chuansha | Catering place | Handling room | Positive | 0 |
| 23 | 5 | chuansha | Catering place | Storage room | Negative | 3 |
| 24 | 5 | chuansha | Catering place | Storage room | Positive | 0 |
| 25 | 5 | chuansha | School | Kitchen | Negative | 4 |
| 26 | 5 | chuansha | School | Kitchen | Positive | 2 |
| 27 | 5 | chuansha | School | Handling room | Negative | 4 |
| 28 | 5 | chuansha | School | Handling room | Positive | 2 |
| 29 | 5 | chuansha | School | Storage room | Negative | 2 |
| 30 | 5 | chuansha | School | Storage room | Positive | 0 |
| 31 | 5 | chuansha | Enterprises and institutions | Kitchen | Negative | 2 |
| 32 | 5 | chuansha | Enterprises and institutions | Kitchen | Positive | 0 |
| 33 | 5 | chuansha | Enterprises and institutions | Handling room | Negative | 0 |
| 34 | 5 | chuansha | Enterprises and institutions | Handling room | Positive | 2 |
| 35 | 5 | chuansha | Enterprises and institutions | Storage room | Negative | 1 |
| 36 | 5 | chuansha | Enterprises and institutions | Storage room | Positive | 0 |
| 37 | 6 | xuanqiao | Catering place | Kitchen | Negative | 6 |
| 38 | 6 | xuanqiao | Catering place | Kitchen | Positive | 0 |
| 39 | 6 | xuanqiao | Catering place | Handling room | Negative | 3 |
| 40 | 6 | xuanqiao | Catering place | Handling room | Positive | 0 |
| 41 | 6 | xuanqiao | Catering place | Storage room | Negative | 3 |
| 42 | 6 | xuanqiao | Catering place | Storage room | Positive | 0 |
| 43 | 6 | xuanqiao | School | Kitchen | Negative | 6 |
| 44 | 6 | xuanqiao | School | Kitchen | Positive | 2 |
| 45 | 6 | xuanqiao | School | Handling room | Negative | 4 |
| 46 | 6 | xuanqiao | School | Handling room | Positive | 1 |
| 47 | 6 | xuanqiao | School | Storage room | Negative | 1 |
| 48 | 6 | xuanqiao | School | Storage room | Positive | 1 |
| 49 | 6 | xuanqiao | Enterprises and institutions | Kitchen | Negative | 1 |
| 50 | 6 | xuanqiao | Enterprises and institutions | Kitchen | Positive | 2 |
| 51 | 6 | xuanqiao | Enterprises and institutions | Handling room | Negative | 1 |
| 52 | 6 | xuanqiao | Enterprises and institutions | Handling room | Positive | 0 |
| 53 | 6 | xuanqiao | Enterprises and institutions | Storage room | Negative | 0 |
| 54 | 6 | xuanqiao | Enterprises and institutions | Storage room | Positive | 1 |
| 55 | 7 | hangtou | Catering place | Kitchen | Negative | 3 |
| 56 | 7 | hangtou | Catering place | Kitchen | Positive | 4 |
| 57 | 7 | hangtou | Catering place | Handling room | Negative | 3 |
| 58 | 7 | hangtou | Catering place | Handling room | Positive | 0 |
| 59 | 7 | hangtou | Catering place | Storage room | Negative | 3 |
| 60 | 7 | hangtou | Catering place | Storage room | Positive | 0 |
| 61 | 7 | hangtou | School | Kitchen | Negative | 6 |
| 62 | 7 | hangtou | School | Kitchen | Positive | 1 |
| 63 | 7 | hangtou | School | Handling room | Negative | 3 |
| 64 | 7 | hangtou | School | Handling room | Positive | 2 |
| 65 | 7 | hangtou | School | Storage room | Negative | 2 |
| 66 | 7 | hangtou | School | Storage room | Positive | 0 |
| 67 | 7 | hangtou | Enterprises and institutions | Kitchen | Negative | 2 |
| 68 | 7 | hangtou | Enterprises and institutions | Kitchen | Positive | 1 |
| 69 | 7 | hangtou | Enterprises and institutions | Handling room | Negative | 2 |
| 70 | 7 | hangtou | Enterprises and institutions | Handling room | Positive | 0 |
| 71 | 7 | hangtou | Enterprises and institutions | Storage room | Negative | 1 |
| 72 | 7 | hangtou | Enterprises and institutions | Storage room | Positive | 0 |
| 73 | 8 | tangqiao | Catering place | Kitchen | Negative | 4 |
| 74 | 8 | tangqiao | Catering place | Kitchen | Positive | 2 |
| 75 | 8 | tangqiao | Catering place | Handling room | Negative | 3 |
| 76 | 8 | tangqiao | Catering place | Handling room | Positive | 2 |
| 77 | 8 | tangqiao | Catering place | Storage room | Negative | 3 |
| 78 | 8 | tangqiao | Catering place | Storage room | Positive | 1 |
| 79 | 8 | tangqiao | School | Kitchen | Negative | 1 |
| 80 | 8 | tangqiao | School | Kitchen | Positive | 3 |
| 81 | 8 | tangqiao | School | Handling room | Negative | 1 |
| 82 | 8 | tangqiao | School | Handling room | Positive | 1 |
| 83 | 8 | tangqiao | School | Storage room | Negative | 1 |
| 84 | 8 | tangqiao | School | Storage room | Positive | 0 |
| 85 | 8 | tangqiao | Enterprises and institutions | Kitchen | Negative | 1 |
| 86 | 8 | tangqiao | Enterprises and institutions | Kitchen | Positive | 1 |
| 87 | 8 | tangqiao | Enterprises and institutions | Handling room | Negative | 1 |
| 88 | 8 | tangqiao | Enterprises and institutions | Handling room | Positive | 0 |
| 89 | 8 | tangqiao | Enterprises and institutions | Storage room | Negative | 1 |
| 90 | 8 | tangqiao | Enterprises and institutions | Storage room | Positive | 0 |
| 91 | 9 | yangsi | Catering place | Kitchen | Negative | 7 |
| 92 | 9 | yangsi | Catering place | Kitchen | Positive | 2 |
| 93 | 9 | yangsi | Catering place | Handling room | Negative | 4 |
| 94 | 9 | yangsi | Catering place | Handling room | Positive | 1 |
| 95 | 9 | yangsi | Catering place | Storage room | Negative | 1 |
| 96 | 9 | yangsi | Catering place | Storage room | Positive | 2 |
| 97 | 9 | yangsi | School | Kitchen | Negative | 4 |
| 98 | 9 | yangsi | School | Kitchen | Positive | 0 |
| 99 | 9 | yangsi | School | Handling room | Negative | 2 |
| 100 | 9 | yangsi | School | Handling room | Positive | 0 |
| 101 | 9 | yangsi | School | Storage room | Negative | 2 |
| 102 | 9 | yangsi | School | Storage room | Positive | 0 |
| 103 | 9 | yangsi | Enterprises and institutions | Kitchen | Negative | 3 |
| 104 | 9 | yangsi | Enterprises and institutions | Kitchen | Positive | 2 |
| 105 | 9 | yangsi | Enterprises and institutions | Handling room | Negative | 1 |
| 106 | 9 | yangsi | Enterprises and institutions | Handling room | Positive | 1 |
| 107 | 9 | yangsi | Enterprises and institutions | Storage room | Negative | 1 |
| 108 | 9 | yangsi | Enterprises and institutions | Storage room | Positive | 0 |
| 109 | 10 | nanmatou | Catering place | Kitchen | Negative | 5 |
| 110 | 10 | nanmatou | Catering place | Kitchen | Positive | 3 |
| 111 | 10 | nanmatou | Catering place | Handling room | Negative | 4 |
| 112 | 10 | nanmatou | Catering place | Handling room | Positive | 1 |
| 113 | 10 | nanmatou | Catering place | Storage room | Negative | 2 |
| 114 | 10 | nanmatou | Catering place | Storage room | Positive | 0 |
| 115 | 10 | nanmatou | School | Kitchen | Negative | 3 |
| 116 | 10 | nanmatou | School | Kitchen | Positive | 2 |
| 117 | 10 | nanmatou | School | Handling room | Negative | 4 |
| 118 | 10 | nanmatou | School | Handling room | Positive | 0 |
| 119 | 10 | nanmatou | School | Storage room | Negative | 2 |
| 120 | 10 | nanmatou | School | Storage room | Positive | 0 |
| 121 | 10 | nanmatou | Enterprises and institutions | Kitchen | Negative | 2 |
| 122 | 10 | nanmatou | Enterprises and institutions | Kitchen | Positive | 0 |
| 123 | 10 | nanmatou | Enterprises and institutions | Handling room | Negative | 2 |
| 124 | 10 | nanmatou | Enterprises and institutions | Handling room | Positive | 0 |
| 125 | 10 | nanmatou | Enterprises and institutions | Storage room | Negative | 1 |
| 126 | 10 | nanmatou | Enterprises and institutions | Storage room | Positive | 0 |
| 127 | 11 | puxing | Catering place | Kitchen | Negative | 5 |
| 128 | 11 | puxing | Catering place | Kitchen | Positive | 3 |
| 129 | 11 | puxing | Catering place | Handling room | Negative | 5 |
| 130 | 11 | puxing | Catering place | Handling room | Positive | 0 |
| 131 | 11 | puxing | Catering place | Storage room | Negative | 4 |
| 132 | 11 | puxing | Catering place | Storage room | Positive | 0 |
| 133 | 11 | puxing | School | Kitchen | Negative | 4 |
| 134 | 11 | puxing | School | Kitchen | Positive | 0 |
| 135 | 11 | puxing | School | Handling room | Negative | 4 |
| 136 | 11 | puxing | School | Handling room | Positive | 0 |
| 137 | 11 | puxing | School | Storage room | Negative | 2 |
| 138 | 11 | puxing | School | Storage room | Positive | 0 |
| 139 | 11 | puxing | Enterprises and institutions | Kitchen | Negative | 0 |
| 140 | 11 | puxing | Enterprises and institutions | Kitchen | Positive | 2 |
| 141 | 11 | puxing | Enterprises and institutions | Handling room | Negative | 0 |
| 142 | 11 | puxing | Enterprises and institutions | Handling room | Positive | 1 |
| 143 | 11 | puxing | Enterprises and institutions | Storage room | Negative | 1 |
| 144 | 11 | puxing | Enterprises and institutions | Storage room | Positive | 0 |
| 145 | 12 | huinan | Catering place | Kitchen | Negative | 5 |
| 146 | 12 | huinan | Catering place | Kitchen | Positive | 3 |
| 147 | 12 | huinan | Catering place | Handling room | Negative | 5 |
| 148 | 12 | huinan | Catering place | Handling room | Positive | 1 |
| 149 | 12 | huinan | Catering place | Storage room | Negative | 4 |
| 150 | 12 | huinan | Catering place | Storage room | Positive | 0 |
| 151 | 12 | huinan | School | Kitchen | Negative | 3 |
| 152 | 12 | huinan | School | Kitchen | Positive | 1 |
| 153 | 12 | huinan | School | Handling room | Negative | 2 |
| 154 | 12 | huinan | School | Handling room | Positive | 0 |
| 155 | 12 | huinan | School | Storage room | Negative | 2 |
| 156 | 12 | huinan | School | Storage room | Positive | 0 |
| 157 | 12 | huinan | Enterprises and institutions | Kitchen | Negative | 2 |
| 158 | 12 | huinan | Enterprises and institutions | Kitchen | Positive | 0 |
| 159 | 12 | huinan | Enterprises and institutions | Handling room | Negative | 1 |
| 160 | 12 | huinan | Enterprises and institutions | Handling room | Positive | 0 |
| 161 | 12 | huinan | Enterprises and institutions | Storage room | Negative | 1 |
| 162 | 12 | huinan | Enterprises and institutions | Storage room | Positive | 0 |
| 163 | 1 | datuan | Catering place | Kitchen | Negative | 5 |
| 164 | 1 | datuan | Catering place | Kitchen | Positive | 5 |
| 165 | 1 | datuan | Catering place | Handling room | Negative | 6 |
| 166 | 1 | datuan | Catering place | Handling room | Positive | 1 |
| 167 | 1 | datuan | Catering place | Storage room | Negative | 6 |
| 168 | 1 | datuan | Catering place | Storage room | Positive | 0 |
| 169 | 1 | datuan | School | Kitchen | Negative | 5 |
| 170 | 1 | datuan | School | Kitchen | Positive | 0 |
| 171 | 1 | datuan | School | Handling room | Negative | 3 |
| 172 | 1 | datuan | School | Handling room | Positive | 0 |
| 173 | 1 | datuan | School | Storage room | Negative | 2 |
| 174 | 1 | datuan | School | Storage room | Positive | 0 |
| 175 | 1 | datuan | Enterprises and institutions | Kitchen | Negative | 2 |
| 176 | 1 | datuan | Enterprises and institutions | Kitchen | Positive | 0 |
| 177 | 1 | datuan | Enterprises and institutions | Handling room | Negative | 1 |
| 178 | 1 | datuan | Enterprises and institutions | Handling room | Positive | 0 |
| 179 | 1 | datuan | Enterprises and institutions | Storage room | Negative | 1 |
| 180 | 1 | datuan | Enterprises and institutions | Storage room | Positive | 0 |
| 181 | 2 | xinchang | Catering place | Kitchen | Negative | 2 |
| 182 | 2 | xinchang | Catering place | Kitchen | Positive | 5 |
| 183 | 2 | xinchang | Catering place | Handling room | Negative | 4 |
| 184 | 2 | xinchang | Catering place | Handling room | Positive | 0 |
| 185 | 2 | xinchang | Catering place | Storage room | Negative | 3 |
| 186 | 2 | xinchang | Catering place | Storage room | Positive | 0 |
| 187 | 2 | xinchang | School | Kitchen | Negative | 4 |
| 188 | 2 | xinchang | School | Kitchen | Positive | 0 |
| 189 | 2 | xinchang | School | Handling room | Negative | 2 |
| 190 | 2 | xinchang | School | Handling room | Positive | 0 |
| 191 | 2 | xinchang | School | Storage room | Negative | 2 |
| 192 | 2 | xinchang | School | Storage room | Positive | 0 |
| 193 | 2 | xinchang | Enterprises and institutions | Kitchen | Negative | 3 |
| 194 | 2 | xinchang | Enterprises and institutions | Kitchen | Positive | 0 |
| 195 | 2 | xinchang | Enterprises and institutions | Handling room | Negative | 0 |
| 196 | 2 | xinchang | Enterprises and institutions | Handling room | Positive | 1 |
| 197 | 2 | xinchang | Enterprises and institutions | Storage room | Negative | 1 |
| 198 | 2 | xinchang | Enterprises and institutions | Storage room | Positive | 0 |
| 199 | 3 | zhoupu | Catering place | Kitchen | Negative | 2 |
| 200 | 3 | zhoupu | Catering place | Kitchen | Positive | 2 |
| 201 | 3 | zhoupu | Catering place | Handling room | Negative | 2 |
| 202 | 3 | zhoupu | Catering place | Handling room | Positive | 0 |
| 203 | 3 | zhoupu | Catering place | Storage room | Negative | 2 |
| 204 | 3 | zhoupu | Catering place | Storage room | Positive | 0 |
| 205 | 3 | zhoupu | School | Kitchen | Negative | 4 |
| 206 | 3 | zhoupu | School | Kitchen | Positive | 0 |
| 207 | 3 | zhoupu | School | Handling room | Negative | 2 |
| 208 | 3 | zhoupu | School | Handling room | Positive | 0 |
| 209 | 3 | zhoupu | School | Storage room | Negative | 1 |
| 210 | 3 | zhoupu | School | Storage room | Positive | 1 |
| 211 | 3 | zhoupu | Enterprises and institutions | Kitchen | Negative | 1 |
| 212 | 3 | zhoupu | Enterprises and institutions | Kitchen | Positive | 1 |
| 213 | 3 | zhoupu | Enterprises and institutions | Handling room | Negative | 1 |
| 214 | 3 | zhoupu | Enterprises and institutions | Handling room | Positive | 0 |
| 215 | 3 | zhoupu | Enterprises and institutions | Storage room | Negative | 1 |
| 216 | 3 | zhoupu | Enterprises and institutions | Storage room | Positive | 0 |

**Database 2**

**Number of cockroaches caught**

| **ID** | **Month** | **Community** | **Place** | **Area** | **Species** | **Development stages** | **Number of cockroaches** |
| --- | --- | --- | --- | --- | --- | --- | --- |
| 1 | 4 | kangqiao | Enterprises and institutions | Kitchen | B. germanica | Nymph | 6 |
| 2 | 4 | kangqiao | Enterprises and institutions | Kitchen | B. germanica | Adult | 10 |
| 3 | 4 | kangqiao | Enterprises and institutions | Storage room | B. germanica | Nymph | 3 |
| 4 | 4 | kangqiao | Enterprises and institutions | Storage room | B. germanica | Adult | 4 |
| 5 | 5 | chuansha | School | Kitchen | P. fuliginosa | Nymph | 14 |
| 6 | 5 | chuansha | School | Kitchen | P. fuliginosa | Adult | 1 |
| 7 | 5 | chuansha | School | Handling room | P. fuliginosa | Nymph | 8 |
| 8 | 5 | chuansha | School | Handling room | P. fuliginosa | Adult | 0 |
| 9 | 5 | chuansha | Enterprises and institutions | Handling room | B. germanica | Nymph | 5 |
| 10 | 5 | chuansha | Enterprises and institutions | Handling room | B. germanica | Adult | 2 |
| 11 | 5 | chuansha | Enterprises and institutions | Handling room | P. fuliginosa | Adult | 2 |
| 12 | 6 | xuanqiao | School | Kitchen | P. fuliginosa | Nymph | 1 |
| 13 | 6 | xuanqiao | School | Kitchen | P. fuliginosa | Adult | 8 |
| 14 | 6 | xuanqiao | School | Handling room | P. fuliginosa | Adult | 1 |
| 15 | 6 | xuanqiao | School | Storage room | P. fuliginosa | Adult | 1 |
| 16 | 6 | xuanqiao | Enterprises and institutions | Kitchen | P. fuliginosa | Nymph | 7 |
| 17 | 6 | xuanqiao | Enterprises and institutions | Kitchen | P. fuliginosa | Adult | 3 |
| 18 | 6 | xuanqiao | Enterprises and institutions | Storage room | P. fuliginosa | Nymph | 2 |
| 19 | 7 | hangtou | Catering place | Kitchen | B. germanica | Adult | 1 |
| 20 | 7 | hangtou | Catering place | Kitchen | P. fuliginosa | Nymph | 7 |
| 21 | 7 | hangtou | Catering place | Kitchen | P. fuliginosa | Adult | 1 |
| 22 | 7 | hangtou | School | Kitchen | P. fuliginosa | Nymph | 2 |
| 23 | 7 | hangtou | School | Kitchen | P. fuliginosa | Adult | 1 |
| 24 | 7 | hangtou | School | Handling room | B. germanica | Nymph | 1 |
| 25 | 7 | hangtou | School | Handling room | P. fuliginosa | Adult | 1 |
| 26 | 7 | hangtou | Enterprises and institutions | Kitchen | P. fuliginosa | Nymph | 2 |
| 27 | 7 | hangtou | Enterprises and institutions | Kitchen | P. fuliginosa | Adult | 1 |
| 28 | 8 | tangqiao | Catering place | Kitchen | B. germanica | Adult | 2 |
| 29 | 8 | tangqiao | Catering place | Handling room | B. germanica | Nymph | 4 |
| 30 | 8 | tangqiao | Catering place | Handling room | B. germanica | Adult | 1 |
| 31 | 8 | tangqiao | Catering place | Storage room | B. germanica | Adult | 2 |
| 32 | 8 | tangqiao | School | Kitchen | P. fuliginosa | Nymph | 12 |
| 33 | 8 | tangqiao | School | Kitchen | P. fuliginosa | Adult | 4 |
| 34 | 8 | tangqiao | School | Handling room | P. fuliginosa | Nymph | 1 |
| 35 | 8 | tangqiao | Enterprises and institutions | Kitchen | P. fuliginosa | Adult | 1 |
| 36 | 9 | yangsi | Catering place | Kitchen | B. germanica | Adult | 2 |
| 37 | 9 | yangsi | Catering place | Handling room | B. germanica | Adult | 1 |
| 38 | 9 | yangsi | Catering place | Storage room | B. germanica | Nymph | 1 |
| 39 | 9 | yangsi | Catering place | Storage room | B. germanica | Adult | 2 |
| 40 | 9 | yangsi | Enterprises and institutions | Kitchen | B. germanica | Nymph | 6 |
| 41 | 9 | yangsi | Enterprises and institutions | Kitchen | B. germanica | Adult | 4 |
| 42 | 9 | yangsi | Enterprises and institutions | Handling room | B. germanica | Adult | 1 |
| 43 | 10 | nanmatou | Catering place | Kitchen | B. germanica | Nymph | 12 |
| 44 | 10 | nanmatou | Catering place | Handling room | B. germanica | Nymph | 2 |
| 45 | 10 | nanmatou | School | Kitchen | B. germanica | Nymph | 5 |
| 46 | 11 | puxing | Catering place | Kitchen | B. germanica | Nymph | 2 |
| 47 | 11 | puxing | Catering place | Kitchen | B. germanica | Adult | 7 |
| 48 | 11 | puxing | Enterprises and institutions | Kitchen | B. germanica | Nymph | 7 |
| 49 | 11 | puxing | Enterprises and institutions | Kitchen | B. germanica | Adult | 1 |
| 50 | 11 | puxing | Enterprises and institutions | Kitchen | P. fuliginosa | Nymph | 1 |
| 51 | 11 | puxing | Enterprises and institutions | Handling room | B. germanica | Nymph | 2 |
| 52 | 11 | puxing | Enterprises and institutions | Handling room | B. germanica | Adult | 1 |
| 53 | 12 | huinan | Catering place | Kitchen | B. germanica | Nymph | 10 |
| 54 | 12 | huinan | Catering place | Kitchen | B. germanica | Adult | 4 |
| 55 | 12 | huinan | Catering place | Handling room | B. germanica | Nymph | 2 |
| 56 | 12 | huinan | Catering place | Handling room | B. germanica | Adult | 1 |
| 57 | 12 | huinan | School | Kitchen | B. germanica | Nymph | 3 |
| 58 | 12 | huinan | School | Kitchen | B. germanica | Adult | 1 |
| 59 | 1 | datuan | Catering place | Kitchen | B. germanica | Nymph | 5 |
| 60 | 1 | datuan | Catering place | Kitchen | B. germanica | Adult | 15 |
| 61 | 1 | datuan | Catering place | Handling room | B. germanica | Adult | 1 |
| 62 | 2 | xinchang | Catering place | Kitchen | B. germanica | Nymph | 3 |
| 63 | 2 | xinchang | Catering place | Kitchen | B. germanica | Adult | 12 |
| 64 | 2 | xinchang | Enterprises and institutions | Handling room | B. germanica | Nymph | 4 |
| 65 | 2 | xinchang | Enterprises and institutions | Handling room | B. germanica | Adult | 2 |
| 66 | 3 | zhoupu | Catering place | Kitchen | B. germanica | Nymph | 11 |
| 67 | 3 | zhoupu | Catering place | Kitchen | B. germanica | Adult | 4 |
| 68 | 3 | zhoupu | School | Storage room | P. fuliginosa | Nymph | 3 |
| 69 | 3 | zhoupu | School | Storage room | P. fuliginosa | Adult | 1 |
| 70 | 3 | zhoupu | Enterprises and institutions | Kitchen | P. fuliginosa | Nymph | 4 |
| 71 | 3 | zhoupu | Enterprises and institutions | Kitchen | P. fuliginosa | Adult | 1 |
