# Supplementary material for: Intestinal pathogens detected in cockroach species within different food-related environment in Pudong, China
Source: Sci Rep. 2024 Jan 23;14:1947. doi: 10.1038/s41598-024-52306-x (PMC10803747; doi:10.1038/s41598-024-52306-x)
Supplement: Supplementary file 3 — Supplementary Information 3. [file 41598_2024_52306_MOESM3_ESM.doc]

**Database 4**

**continue**

| **year** | **month** | **sample** | **positive** | **rate** | **Sapovirus** | **Norovirus** | **Astrovirus** | **Adenovirus** | **Rotavirus** | ***E. coli*** | ***Aeromonas hydrophila*** |
| --- | --- | --- | --- | --- | --- | --- | --- | --- | --- | --- | --- |
| 2021 | 1 | 22 | 12 | 54.55 | 0 | 8 | 0 | 0 | 4 | 0 | 0 |
|  | 2 | 22 | 18 | 81.82 | 0 | 3 | 0 | 1 | 14 | 3 | 0 |
|  | 3 | 26 | 13 | 50.00 | 4 | 6 | 1 | 1 | 1 | 1 | 0 |
|  | 4 | 42 | 11 | 26.19 | 1 | 8 | 0 | 2 | 0 | 3 | 0 |
|  | 5 | 52 | 15 | 28.85 | 0 | 9 | 2 | 1 | 3 | 4 | 0 |
|  | 6 | 60 | 8 | 13.33 | 2 | 1 | 4 | 1 | 0 | 7 | 0 |
|  | 7 | 86 | 24 | 27.91 | 1 | 5 | 11 | 6 | 1 | 12 | 0 |
|  | 8 | 57 | 7 | 12.28 | 0 | 0 | 4 | 3 | 0 | 13 | 0 |
|  | 9 | 31 | 3 | 9.68 | 0 | 0 | 0 | 0 | 3 | 3 | 0 |
|  | 10 | 22 | 7 | 31.82 | 0 | 5 | 0 | 0 | 2 | 4 | 0 |
|  | 11 | 26 | 11 | 42.31 | 1 | 6 | 0 | 2 | 2 | 2 | 0 |
|  | 12 | 40 | 31 | 77.50 | 1 | 11 | 7 | 11 | 1 | 2 | 0 |

**continued table**

| *STEC* | Nontyphoid Salmonella | *Vibrio parahaemolyticus* | Shiga-like Orthomonas | *Campylobacter jejuni* | *Yersinia enterocolitica* |
| --- | --- | --- | --- | --- | --- |
| 0 | 0 | 0 | 0 | 1 | 0 |
| 0 | 0 | 0 | 0 | 0 | 0 |
| 0 | 0 | 0 | 0 | 0 | 0 |
| 0 | 2 | 0 | 0 | 0 | 0 |
| 2 | 2 | 0 | 0 | 0 | 0 |
| 0 | 5 | 1 | 0 | 0 | 0 |
| 0 | 6 | 2 | 0 | 0 | 1 |
| 0 | 14 | 0 | 2 | 0 | 0 |
| 0 | 1 | 5 | 0 | 0 | 0 |
| 0 | 1 | 1 | 0 | 0 | 0 |
| 0 | 0 | 0 | 0 | 0 | 0 |
| 0 | 0 | 0 | 0 | 1 | 0 |
